# Cemiplimab monotherapy in Japanese patients with recurrent or metastatic cervical cancer

**DOI:** 10.1002/cam4.70236

**Published:** 2024-09-26

**Authors:** Kosei Hasegawa, Shunji Takahashi, Kimio Ushijima, Masao Okadome, Kan Yonemori, Harushige Yokota, Ignace Vergote, Bradley J. Monk, Krishnansu S. Tewari, Keiichi Fujiwara, Jingjin Li, Shaheda Jamil, Anne Paccaly, Kazuhiro Takehara, Tomoka Usami, Yoichi Aoki, Nao Suzuki, Yoichi Kobayashi, Yoshio Yoshida, Hidemichi Watari, Frank Seebach, Israel Lowy, Melissa Mathias, Matthew G. Fury, Ana Oaknin

**Affiliations:** ^1^ Department of Gynecologic Oncology Saitama Medical University International Medical Center Hidaka Saitama Japan; ^2^ Department of Medical Oncology Cancer Institute Hospital of Japanese Foundation for Cancer Research Tokyo Japan; ^3^ Department of Obstetrics and Gynecology Kurume University School of Medicine Kurume Japan; ^4^ Gynecology Service National Hospital Organization Kyushu Cancer Center Fukuoka Japan; ^5^ Department of Breast and Medical Oncology National Cancer Center Hospital Tokyo Japan; ^6^ Saitama Cancer Center Saitama Japan; ^7^ Department of Gynecology and Obstetrics University Hospitals, Katholieke Universiteit Leuven Leuven Belgium; ^8^ Division of Gynecologic Oncology University of Arizona and Creighton University Phoenix Arizona USA; ^9^ Department of Gynecology and Obstetrics University of California Irvine California USA; ^10^ Regeneron Pharmaceuticals, Inc. Tarrytown New York USA; ^11^ Department of Gynaecologic Oncology National Hospital Organization Shikoku Cancer Center Matsuyama Japan; ^12^ Department of Obstetrics and Gynaecology Ehime University School of Medicine Toon Japan; ^13^ Department of Obstetrics and Gynaecology University of the Ryukyus Hospital Okinawa Japan; ^14^ Department of Obstetrics and Gynaecology St Marianna University School of Medicine Kawasaki Japan; ^15^ Department of Obstetrics and Gynaecology, Faculty of Medicine Kyorin University Tokyo Japan; ^16^ Department of Obstetrics and Gynaecology University of Fukui Fukui Japan; ^17^ Department of Obstetrics and Gynaecology Hokkaido University Graduate School of Medicine Sapporo Japan; ^18^ Gynaecologic Cancer Programme, Vall d'Hebron Institute of Oncology (VHIO) Hospital Universitari Vall d'Hebron, Vall d'Hebron Barcelona Hospital Campus Barcelona Spain

**Keywords:** cemiplimab, cervical cancer, chemotherapy, immunotherapy, programmed cell death‐1

## Abstract

**Background:**

In the phase 3 EMPOWER‐Cervical 1/GOG‐3016/ENGOT‐cx9 study, cemiplimab significantly improved overall survival (OS) versus chemotherapy for patients with recurrent or metastatic cervical cancer who progressed after first‐line platinum‐based chemotherapy. We present a post hoc subgroup analysis of patients enrolled in Japan.

**Methods:**

Patients were enrolled regardless of programmed cell death‐ligand 1 status and randomized 1:1 to cemiplimab 350 mg intravenously every 3 weeks or investigator's choice  single‐agent chemotherapy for up to 96 weeks. Primary endpoint was OS. Key secondary endpoints were progression‐free survival (PFS) and objective response rate (ORR).

**Results:**

Overall, 608 patients were randomized, of whom 56 (9.2%) were in Japan (cemiplimab, *n* = 29; chemotherapy, *n* = 27). The median (range) duration of follow‐up was 13.6 (6.0–25.3) versus 18.2 (6.0–38.2) months for patients in Japan and for the overall population, respectively. Median OS (95% confidence interval [CI]) was 8.4 (7.0‐not evaluable) and 9.4 (5.4–14.9) months for cemiplimab versus chemotherapy (hazard ratio [HR]: 0.86; 95% CI: 0.43–1.68). Median PFS (95% CI) was 4.0 (1.4–8.2) versus 3.7 (1.8–4.2) months with cemiplimab and chemotherapy (HR: 0.90; 95% CI: 0.50–1.61), respectively. ORR was 17.2% for cemiplimab and 7.4% for chemotherapy (odds ratio, 2.47; 95% CI, 0.44–13.99). Incidence of treatment‐emergent adverse events at any grade was 79.3% for cemiplimab and 100% for chemotherapy. Grade ≥3 adverse events were 37.9% versus 66.7% with cemiplimab and chemotherapy, respectively.

**Discussion:**

While acknowledging limitations inherent to a small subgroup analysis, the HR of 0.86 observed in Japanese patients suggests an emerging survival benefit despite a 4.6‐month shorter median duration of follow‐up versus the overall study population.

## 1 INTRODUCTION

Cervical cancer ranks as the fourth most diagnosed cancer and is one of the leading causes of cancer‐related deaths among women. Globally, there were about 604,127 new cases and 341,831 deaths in 2020.^1^ In Japan, there is an increased incidence of cervical cancer with approximately 12,785 new cases and 4213 deaths in 2020.^2^ This estimates an annual age‐standardized incidence rate of 15.2 per 100,000 women, which is higher than the estimates for the global population (13.3 per 100,000 women) and other high‐income countries in East Asia (China: 10.7 per 100,000 women; Republic of Korea: 8.1 per 100,000 women).^2^ Additionally, there has been an increase in both age‐adjusted incidence and the mortality rate of cervical cancer in Japanese women in the last 2 decades.^3^, ^4^

In 99.7% of cervical cancer cases, the etiology is infection with high‐risk oncogenic subsets of human papillomavirus (HPV).^5^ Organized screening programs using cervical cytology and DNA testing for specific HPV subsets, and the availability of prophylactic vaccines based on virus‐like particle technology, have reduced the incidence of cervical cancer. However, globally, many women lack access to screening programs, prophylactic vaccines, and high‐quality interventional treatments when required.^6^, ^7^, ^8^, ^9^, ^10^ Japan has a low HPV and cervical screening uptake. The vaccination program started in 2010 and was suspended a couple of years later due to vaccine hesitancy; it has only recently been restarted. These factors together contribute to this public health concern.^11^, ^12^

The standard therapy for early invasive disease (International Federation of Gynecology and Obstetrics [FIGO] stage IB_1_‐IB_2_)^13^ is radical hysterectomy with bilateral pelvic lymph node dissection with or without tailored adjuvant therapy.^14^ For locally advanced disease (FIGO stage IB_3_‐IVA),^13^ patients are treated with platinum‐based chemoradiation and high dose‐rate intracavitary brachytherapy.^14^, ^15^ For patients with local recurrence where pelvic exenteration with urinary diversion is not an option, and those with metastatic disease (FIGO stage IVB),^13^ the standard treatment is platinum‐based chemotherapy^16^, ^17^ with or without bevacizumab (an anti‐angiogenic agent).^18^ In recurrent or metastatic disease, first‐line treatment with platinum‐based chemotherapy with or without bevacizumab was associated with a median overall survival (OS) of 13.3–18.3 months.^17^, ^18^ Patients usually experience disease progression on this treatment regimen.^11^ In Japan, irinotecan has been used to treat cervical cancer since 1994 and is still used in combination treatment regimens.^19^, ^20^ While irinotecan has shown clinical benefit, there is a risk of severe leukopenia, thrombocytopenia, and diarrhea in some patients^21^; due to this, it is not recommended for all patients and requires rigorous screening for contraindications beforehand.

More recently the combination of pembrolizumab, a programmed cell death‐1 (PD‐1) inhibitor, with platinum‐based chemotherapy with or without bevacizumab has been approved in Japan, Europe, and the United States as first‐line treatment for patients with metastatic cervical cancer based on the results of the KEYNOTE‐826 study (ClinicalTrials.gov identifier NCT03635567).^22^, ^23^, ^24^, ^25^, ^26^ Pembrolizumab, when added to platinum‐based chemotherapy with or without bevacizumab, showed prolonged survival rates compared to chemotherapy alone in the KEYNOTE‐826 study.^26^ Notably, in Japan, first‐line treatment with pembrolizumab for metastatic cervical cancer is not limited by programmed cell death‐ligand 1 (PD‐L1) expression status, unlike in Europe and the United States where pembrolizumab is only approved for patients with PD‐L1 expression ≥ 1%.^22^, ^23^, ^24^

Cemiplimab is a highly potent, fully human, hinge‐stabilized monoclonal antibody targeting PD‐1, generated using VelocImmune® technology.^27^, ^28^, ^29^ In the international phase 3 EMPOWER‐Cervical 1/Gynecologic Oncology Group (GOG)‐3016/European Network of Gynecological Oncological Trial groups (ENGOT)‐cx9 study (ClinicalTrials.gov identifier NCT03257267), cemiplimab was compared with single‐agent chemotherapy based on the choice of the investigator in 608 patients with cervical cancer who had disease progression after first‐line treatment with platinum‐based chemotherapy for recurrence or metastases.^30^ Patients were enrolled irrespective of PD‐L1 expression status, including those with squamous cell carcinoma (SCC) or adenocarcinoma/adenosquamous carcinoma (AC) histologic subtypes.^30^ The median OS was significantly longer with cemiplimab than with chemotherapy (12.0 months vs 8.5 months; hazard ratio [HR] for death: 0.69; 95% confidence interval [CI]: 0.56–0.84; 1‐sided *p* < 0.001) in the overall study population.^30^

Based on the EMPOWER‐Cervical 1/GOG‐3016/ENGOT‐cx9 study, cemiplimab monotherapy was approved in Japan, Brazil, Canada, and Europe for the second‐line setting in patients with recurrent or metastatic cervical cancer regardless of tumor histology or PD‐L1 presentation.^31^, ^32^, ^33^, ^34^

Ethnicity should be carefully considered when designing a clinical study, as clinical response, safety, and tolerability with systemic therapy may vary between Asian and non‐Asian patients.^35^, ^36^ Subgroup analyses based on race and ethnicity are crucial in understanding the differential responses to cancer therapies, given the genetic, environmental, and lifestyle factors that can influence treatment outcomes.^37^, ^38^, ^39^ Data from clinical trials suggest that Asian patients have comparable or improved efficacy with PD‐1/PD‐L1 inhibitors when compared to non‐Asian patients.^40^, ^41^, ^42^, ^43^ A subgroup analysis from the phase 3 KEYNOTE‐826 study including Japanese patients with metastatic cervical cancer indicated prolonged OS and progression‐free survival (PFS) with pembrolizumab compared to chemotherapy, consistent with findings from the overall study population.^11^, ^26^ A meta‐analysis comprising 11,020 patients from 19 randomized clinical trials of PD‐1/PD‐L1 inhibitors demonstrated that Asian patients with metastatic solid tumors had a greater OS and PFS benefit than non‐Asian patients.^36^

Of 608 patients enrolled in the EMPOWER‐Cervical 1/GOG‐3016/ENGOT‐cx9 study, 56 (9.2%) were from Japan. Thus, we performed a post hoc subgroup analysis to evaluate the efficacy, safety, and pharmacokinetics of cemiplimab in the Japanese subgroup, and to assess whether the results from this subgroup were similar to those reported for the overall EMPOWER‐Cervical 1/GOG‐3016/ENGOT‐cx9 population.

## 2 MATERIALS AND METHODS

### 2.1 Study oversight

EMPOWER‐Cervical 1/GOG‐3016/ENGOT‐cx9, an open‐label, multicenter, phase 3 study, was sponsored by Regeneron Pharmaceuticals, Inc., and Sanofi, which provided cemiplimab without charge according to ENGOT‐GOG Model C.^44^ The study protocol and all amendments were approved by the appropriate institutional review board or independent ethics committee at each participating study site. The study was conducted in accordance with the principles of the Declaration of Helsinki and the International Conference on Harmonization Good Clinical Practice guidelines. All patients provided written informed consent before enrollment.

### 2.2 Patients

The EMPOWER‐Cervical 1/GOG‐3016/ENGOT‐cx9 study recruited 608 patients at approximately 100 sites in 14 countries (Australia, *n* = 30; Belgium, *n* = 15; Brazil, *n* = 89; Canada, *n* = 42; Greece, *n* = 9; Italy, *n* = 35; Japan, *n* = 56; the Republic of Korea, *n* = 76; Poland, *n* = 45; Russia, *n* = 85; Spain, *n* = 66; Taiwan, *n* = 34; the United Kingdom, *n* = 2; and the United States, *n* = 24). This subgroup analysis evaluated the efficacy, safety, and pharmacokinetics of cemiplimab in the Japanese patient population enrolled in EMPOWER‐Cervical 1/GOG‐3016/ENGOT‐cx9.

The detailed methodology for the full study was published previously.^30^ All patients had recurrent or metastatic cervical cancer which had progressed after first‐line platinum‐based chemotherapy, without any curative‐intent option. Patients must have received prior bevacizumab and prior paclitaxel therapy unless they had refused, were deemed unsuitable, or did not have access to bevacizumab. Patients were required to have an Eastern Cooperative Oncology Group (ECOG) performance status of 0 or 1^45^ and measurable disease based on Response Evaluation Criteria in Solid Tumors (RECIST) version 1.1.^46^ SCC or AC histologic subtypes were permitted, regardless of PD‐L1 expression status.

### 2.3 Study design

Patients were randomized (1:1) to receive either cemiplimab at a fixed dose of 350 mg every 3 weeks (Q3W) intravenously as a 30‐minute infusion or the investigator's choice of single‐agent chemotherapy. The investigator selected single‐agent chemotherapy prior to randomization from protocol‐specified options, which comprised pemetrexed (500 mg/m^2^ administered intravenously Q3W), topotecan (1 mg/m^2^ administered intravenously daily for 5 days Q3W), irinotecan (100 mg/m^2^ administered intravenously weekly for 4 weeks every 6 weeks), gemcitabine (1000 mg/m^2^ administered intravenously on days 1 and 8 Q3W), or vinorelbine (30 mg/m^2^ administered intravenously on days 1 and 8 Q3W). Patients in both treatment groups were treated for up to 96 weeks in 6‐week treatment cycles, until disease progression or development of unacceptable toxicity. Due to the potential for unconventional responses (i.e., pseudoprogression) with immune checkpoint inhibitors, patients in the cemiplimab treatment group were permitted to be treated after disease progression as long as there was no deterioration of ECOG performance status, evidence of rapid disease progression, or severe adverse events (AEs) requiring permanent discontinuation of cemiplimab.

### 2.4 Outcomes

The primary endpoint was OS, defined as the time from randomization until death due to any cause. Secondary endpoints included PFS, defined as the time from randomization to the date of the first documented tumor progression according to RECIST version 1.1 or death from any cause, objective response rate (ORR), calculated as the number of patients with a best overall response of confirmed complete response or partial response divided by the number of patients in the efficacy analysis set, and the safety of cemiplimab versus chemotherapy. Exploratory endpoints included pharmacokinetics, measured by cemiplimab concentrations in serum, and immunogenicity, evaluated by measuring the presence of antidrug antibodies (ADAs) in serum.

### 2.5 Assessments

Radiographic imaging of target lesions was performed at screening and on day 42 in cycles 1–4, 6, 8, 10, 12, 14, and 16. RECIST version 1.1.^46^ was used to assess tumor response. Safety was assessed based on the occurrence and severity of AEs graded according to the National Cancer Institute Common Terminology Criteria for Adverse Events version 4.03.^47^ To measure cemiplimab concentrations, blood samples were obtained from patients before dosing and within 10 minutes after the end of the infusion on day 1 of cycles 1–7, 9, 11, 13, and 15. Functional cemiplimab (reported elsewhere as cemiplimab) concentrations in serum were measured using a validated enzyme‐linked immunosorbent assay with a lower limit of quantification of 0.078 mg/L. Blood samples to measure ADAs against cemiplimab and neutralizing anti‐cemiplimab antibodies in serum were collected before dosing on day 1 of cycles 1, 3, 7, 11, and 15. ADAs against cemiplimab in serum were measured using a validated electrochemiluminescence bridging immunoassay. A validated competitive ligand‐binding assay evaluated the presence of neutralizing antibodies in serum samples that were ADA positive.

### 2.6 Statistical analysis

OS, PFS, and ORR were assessed in all randomized patients grouped as per treatment assignment, irrespective of adherence. Safety was assessed in all randomized patients who received ≥1 dose of the assigned treatment. The pharmacokinetics analysis population comprised all randomized patients who received at least 1 dose of cemiplimab and who had at least 1 non‐missing concentration of cemiplimab following administration of the initial dose. The ADA analysis population comprised all treated patients who received at least 1 dose of cemiplimab and had at least 1 non‐missing anti‐cemiplimab antibody result following administration of the initial dose.

OS and PFS were evaluated by the stratified log‐rank test using tumor histology as a stratification factor for the Japanese subgroup, and geographic region and tumor histology for the overall study population. Kaplan–Meier method was used to obtain the estimated median OS and PFS.^48^ HRs and associated 95% CIs were estimated by stratified Cox proportional‐hazards models.^49^ ORR and associated odds ratios (ORs) were analyzed by the Cochran–Mantel–Haenszel test^50^, ^51^ using tumor histology as a stratification factor for the Japanese subgroup, and geographic region and tumor histology as stratification factors for the overall study population. ORRs and associated 95% CIs were calculated using the Clopper‐Pearson method.^52^ As this was a subgroup analysis, statistical tests for treatment comparisons were not performed for the Japanese subgroup.

## 3 RESULTS

### 3.1 Patients and treatments

Of 608 patients enrolled in the EMPOWER‐Cervical 1/GOG‐3016/ENGOT‐cx9 study between September 2017 and August 2020, 56 (9.2%) patients from 13 sites in Japan were randomized to receive cemiplimab (*n* = 29) or the investigator's choice of chemotherapy (*n* = 27). Of those randomized to chemotherapy, 19 (70.4%) patients were assigned to receive gemcitabine and 8 (29.6%) patients were assigned to receive irinotecan (Table S1). The median age in the Japanese subgroup was 57.0 years (range: 30–81) versus 51.0 years (range: 22–87) in the overall study population (Table 1). The median body mass index (BMI) of Japanese patients was lower than the overall study population at 21.3 kg/m^2^ (range: 15.0–36.8) versus 23.6 kg/m^2^ (range: 14.2–49.0). In the Japanese subgroup, 94.6% (*n* = 53) of patients had SCC and 5.4% (*n* = 3) of patients had AC. In the overall study population, 77.8% (*n* = 473) of patients had SCC and 22.2% (*n* = 135) of patients had AC.

**TABLE 1:** Baseline demographic and disease characteristics^a^.

Characteristic	Overall study population			Japanese subgroup		
	Cemiplimab (*n* = 304)	Chemotherapy (*n* = 304)	Overall (*N* = 608)	Cemiplimab (*n* = 29)	Chemotherapy (*n* = 27)	Overall (*N* = 56)
Age						
Median (range), years	51.0 (22–81)	50.0 (24–87)	51.0 (22–87)	57.0 (34–78)	52.0 (30–81)	57.0 (30–81)
<65 years, *n* (%)	269 (88.5)	264 (86.8)	533 (87.7)	19 (65.5)	19 (70.4)	38 (67.9)
Body weight, median (range), kg	61.8 (35.9–128.7)	61.2 (35.0–120.0)	61.6 (35.0–128.7)	52.7 (35.9–84.2)	50.0 (36.6–81.7)	51.5 (35.9–84.2)
Body mass index, median (range) kg/m^2^	23.5 (14.2–49.0)	23.7 (14.2–46.6)	23.6 (14.2–49.0)	21.5 (15.9–36.8)	19.8 (15.0–32.7)	21.3 (15.0–36.8)
Region of enrollment, *n* (%)						
Asia	83 (27.3)	83 (27.3)	166 (27.3)	29 (100)	27 (100)	56 (100)
North America	32 (10.5)	34 (11.2)	66 (10.9)	NA	NA	NA
Rest of the world	189 (62.2)	187 (61.5)	376 (61.8)	NA	NA	NA
ECOG performance status, *n* (%)						
0	142 (46.7)	141 (46.4)	283 (46.5)	22 (75.9)	19 (70.4)	41 (73.2)
1	162 (53.3)	163 (53.6)	325 (53.5)	7 (24.1)	8 (29.6)	15 (26.8)
Histology/cytology, *n* (%)						
SCC	240 (78.9)	233 (76.6)	473 (77.8)	27 (93.1)	26 (96.3)	53 (94.6)
AC	64 (21.1)	71 (23.4)	135 (22.2)	2 (6.9)	1 (3.7)	3 (5.4)
Extent of disease, *n* (%)						
Metastatic	284 (93.4)	290 (95.4)	574 (94.4)	28 (96.6)	24 (88.9)	52 (92.9)
Recurrent or persistent	20 (6.6)	14 (4.6)	34 (5.6)	1 (3.4)	3 (11.1)	4 (7.1)
Prior lines of therapy for recurrent or metastatic disease, *n* (%)						
1	177 (58.2)	169 (55.6)	346 (56.9)	12 (41.4)	12 (44.4)	24 (42.9)
>1	124 (40.8)	135 (44.4)	259 (42.6)	17 (58.6)	15 (55.6)	32 (57.1)
Prior bevacizumab use, *n* (%)^b^						
Yes	148 (48.7)	149 (49.0)	297 (48.8)	18 (62.1)	15 (55.6)	33 (58.9)
No	156 (51.3)	155 (51.0)	311 (51.2)	11 (37.9)	12 (44.4)	23 (41.1)
Prior paclitaxel use, *n* (%)						
Yes	273 (89.8)	287 (94.4)	560 (92.1)	29 (100)	26 (96.3)	55 (98.2)
No	31 (10.2)	17 (5.6)	48 (7.9)	0	1 (3.7)	1 (1.8)
Prior lines of cancer systemic therapy, *n* (%)						
1	81 (26.6)	66 (21.7)	147 (24.2)	3 (10.3)	2 (7.4)	5 (8.9)
>1	223 (73.4)	238 (78.3)	461 (75.8)	26 (89.7)	25 (92.6)	51 (91.1)
Number of patients with any prior cancer‐related radiotherapy, *n* (%)	262 (86.2)	259 (85.2)	521 (85.7)	25 (86.2)	24 (88.9)	49 (87.5)

Abbreviations: AC, adenocarcinoma or adenosquamous carcinoma; ECOG, Eastern Cooperative Oncology Group; NA, not applicable; SCC, squamous cell carcinoma.

^a^ Data cutoff date: January 4, 2021.

^b^ Based on interactive web response system data.

The proportion of patients who had received >1 prior line of systemic therapy for recurrent disease was numerically higher among the Japanese subgroup (57.1%, *n* = 32) than the overall study population (42.6%, *n* = 259). Similarly, the proportion of patients with prior bevacizumab use was numerically higher among the Japanese subgroup (58.9%, *n* = 33) than the overall study population (48.8%, *n* = 297). Baseline characteristics were generally comparable between the 2 treatment groups in both the Japanese subgroup and the overall study population (Table 1). All data reported are based on a data cutoff date of January 4, 2021.

Median duration of patient follow‐up from treatment randomization to data cutoff was 13.6 months (range: 6.0–25.3) in the Japanese subgroup and 18.2 months (range: 6.0–38.2) in the overall study population. In the Japanese subgroup, median duration of treatment exposure was 18.1 weeks (range: 3.0–71.9) with cemiplimab and 11.0 weeks (range: 1.0–35.0) with chemotherapy. The median duration of treatment exposure with cemiplimab was 15.2 weeks (range: 1.4–100.7) and 10.1 weeks (range: 1.0–81.9) with chemotherapy in the overall study population.

### 3.2 Efficacy

**FIGURE 1:**
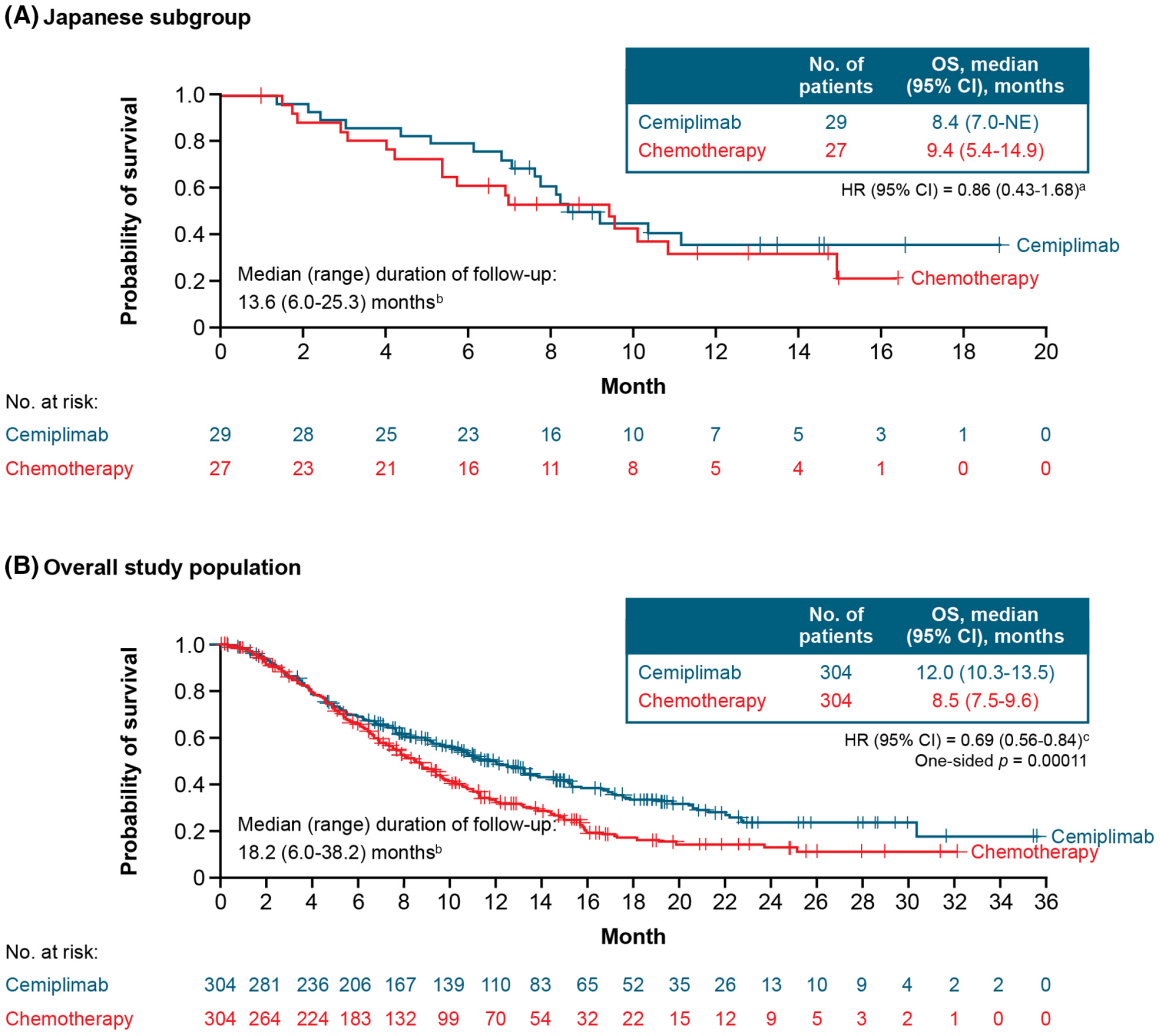
Overall survival in (A) the Japanese subgroup and (B) the overall population. Data cutoff date: January 4, 2021. ^a^Stratified by histology (SCC vs. AC) according to IWRS. ^b^From randomization to data cutoff date. ^c^Stratified by histology (SCC vs. AC) and geographic region (North America vs. Asia vs. ROW) according to IWRS. AC, adenocarcinoma or adenosquamous carcinoma; CI, confidence interval; HR, hazard ratio; IWRS, interactive web response system; OS, overall survival; ROW, rest of world; SCC, squamous cell carcinoma.

In the Japanese subgroup, median OS was 8.4 months (95% CI: 7.0‐not evaluable) with cemiplimab versus 9.4 months (95% CI: 5.4–14.9) with chemotherapy (HR: 0.86; 95% CI: 0.43–1.68) (Figure 1A). In the overall trial population, median OS was 12.0 months (95% CI: 10.3–13.5) with cemiplimab versus 8.5 months (95% CI: 7.5–9.6) with chemotherapy (HR: 0.69; 95% CI: 0.56–0.84; 1‐sided *p* < 0.001) (Figure 1B).

**FIGURE 2:**
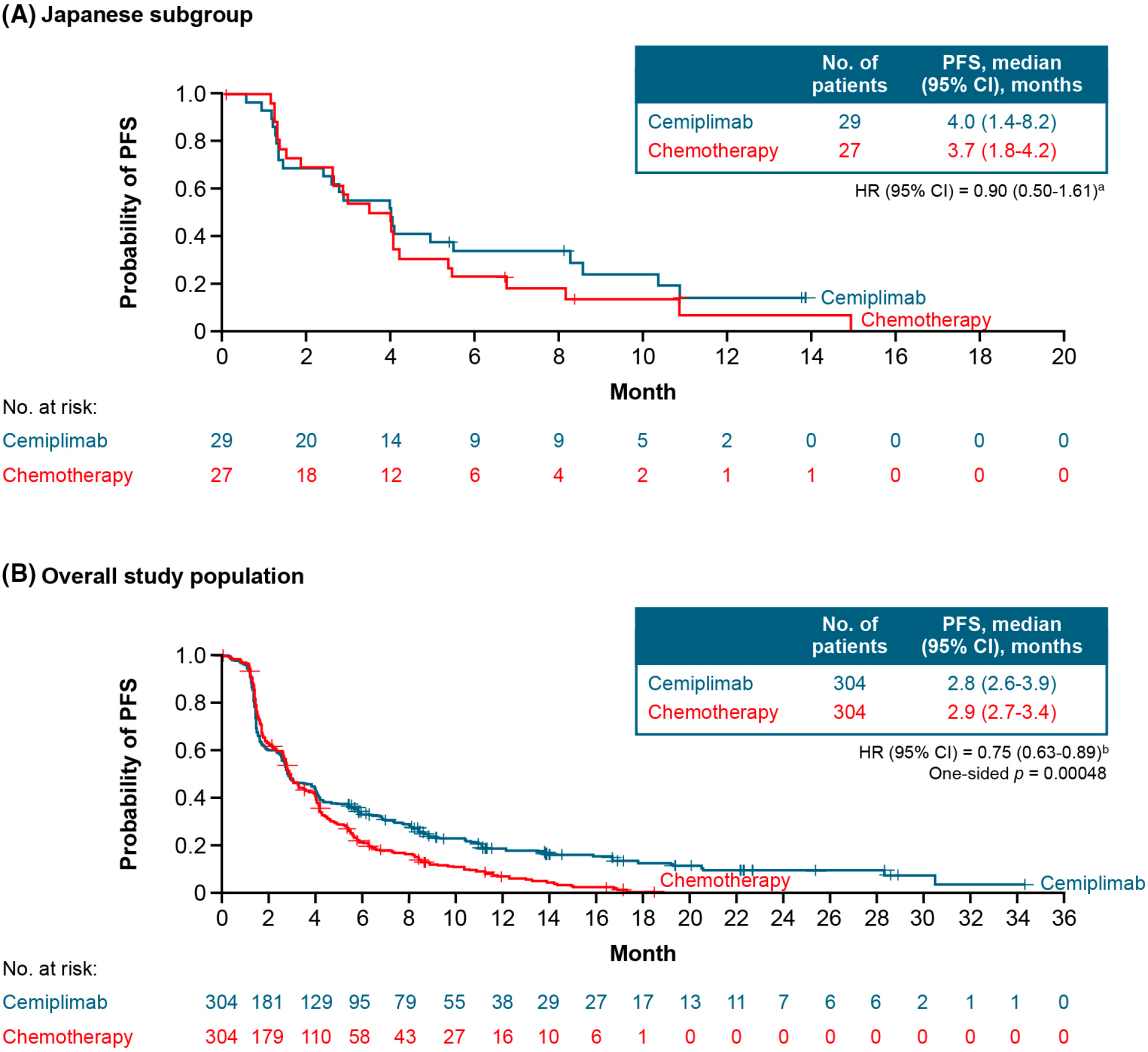
Progression‐free survival in (A) the Japanese subgroup and (B) the overall population. Data cutoff date: January 4, 2021. ^a^Stratified by histology (SCC vs. AC) according to IWRS. ^b^Stratified by histology (SCC vs. AC) and geographic region (North America vs. Asia vs. ROW) according to IWRS. AC, adenocarcinoma or adenosquamous carcinoma; CI, confidence interval; HR, hazard ratio; IWRS, interactive web response system; PFS, progression‐free survival; ROW, rest of world; SCC, squamous cell carcinoma.

In the Japanese subgroup, median PFS was 4.0 months (95% CI: 1.4–8.2) with cemiplimab and 3.7 months (95% CI: 1.8–4.2) with chemotherapy (HR: 0.90; 95% CI: 0.50–1.61) (Figure 2A). In the overall trial population, median PFS was 2.8 months (95% CI: 2.6–3.9) with cemiplimab and 2.9 months (95% CI: 2.7–3.4) with chemotherapy (HR: 0.75; 95% CI: 0.63–0.89; 1‐sided *p* < 0.001) (Figure 2B).

In the Japanese subgroup, ORR was higher with cemiplimab at 17.2% (95% CI: 5.8–35.8) than with chemotherapy at 7.4% (95% CI: 0.9–24.3) (OR: 2.47; 95% CI: 0.44–13.99) (Table 2). Due to the number of responders (cemiplimab, *n* = 5; chemotherapy, *n* = 2) among patients enrolled in Japan, it was not possible to perform a Kaplan–Meier estimation of duration of response.

**TABLE 2:** Tumor response per RECIST version 1.1 by investigator assessment^a^.

Variable	Overall study population		Japanese subgroup	
	Cemiplimab (*n* = 304)	Chemotherapy (*n* = 304)	Cemiplimab (*n* = 29)	Chemotherapy (*n* = 27)
Objective response				
ORR (complete response + partial response), *n* (%)	50 (16.4)	19 (6.3)	5 (17.2)	2 (7.4)
95% CI for ORR^b^	12.5–21.1	3.8–9.6	5.8–35.8	0.9–24.3
Best overall tumor response, *n* (%)				
Complete response^c^	10 (3.3)	3 (1.0)	1 (3.4)	1 (3.7)
Partial response^c^	40 (13.2)	16 (5.3)	4 (13.8)	1 (3.7)
Stable disease^d^	125 (41.1)	148 (48.7)	15 (51.7)	15 (55.6)
Progressive disease	105 (34.5)	88 (28.9)	9 (31.0)	7 (25.9)
Not evaluable	24 (7.9)	49 (16.1)	0	3 (11.1)
Stratified Cochran–Mantel–Haenszel test 1‐sided *p* value^e^	<0.001		NP	
Odds ratio (95% CI)^e^	2.984 (1.707–5.215)		2.471 (0.436–13.990)	
Kaplan–Meier estimated duration of response, median (95% CI), months^f^, ^g^	16.4 (12.4‐NE)	6.9 (5.1–7.7)	NP	NP

Abbreviations: CI, confidence interval; NE, not evaluable; NP, not performed; ORR, objective response rate; RECIST, Response Evaluation Criteria in Solid Tumors.

^a^ Data cutoff date: January 4, 2021.

^b^ Clopper‐Pearson exact CI.

^c^ Complete response and partial response must have been confirmed by repeated assessments ≥4 weeks apart.

^d^ Stable disease criteria must have been met at least once for ≥4 weeks after the first dose date.

^e^ For patients enrolled in Japan, odds ratio was determined using histology stratified by the Cochran–Mantel–Haenszel test. Statistical tests were not performed for the Japanese subgroup. For the overall study population, 1‐sided *p* values and odds ratios were determined using geographic region and histology stratified by the Cochran–Mantel–Haenszel test. Due to the low response rate in the chemotherapy group, the results from the Cochran–Mantel–Haenszel test should be interpreted with caution.

^f^ Based on patients with confirmed complete response or partial response.

^g^ Due to the number of responders (cemiplimab, *n* = 5; chemotherapy, *n* = 2) among patients enrolled in Japan, it was not possible to perform a Kaplan–Meier estimation of duration of response.

In the overall population, ORR was higher with cemiplimab at 16.4% (95% CI: 12.5–21.1) than with chemotherapy at 6.3% (95% CI: 3.8–9.6) (OR: 2.98; 95% CI: 1.71–5.22; 1‐sided *p* < 0.001). The Kaplan–Meier estimated median duration of response in the overall population was 16.4 months (95% CI: 12.4‐not evaluable) with cemiplimab and 6.9 months (95% CI: 5.1–7.7) with chemotherapy (Table 2).

### 3.3 Safety

The safety profiles of cemiplimab and the investigator's of choice chemotherapy in the Japanese subgroup and the overall population are summarized in Table 3. In the Japanese subgroup, treatment‐emergent AEs (TEAEs) of any grade, regardless of attribution, occurred in 79.3% (*n* = 23) of patients treated with cemiplimab and 100% (*n* = 27) of patients treated with chemotherapy; the events were Grade ≥3 in 37.9% (*n* = 11) and 66.7% (*n* = 18) of patients, respectively (Table 3). Among the overall population, TEAEs of any grade, regardless of attribution, occurred in 88.3% (*n* = 265) of patients treated with cemiplimab and 91.4% (*n* = 265) of patients treated with chemotherapy; the events were Grade ≥3 in 45.0% (*n* = 135) and 53.4% (*n* = 155) of patients, respectively (Table 3).

**TABLE 3:** Summary of AEs^a^.

	Overall study population				Japanese subgroup			
	Cemiplimab (*n* = 300)		Chemotherapy (*n* = 290)		Cemiplimab (*n* = 29)		Chemotherapy (*n* = 27)	
Event	Any grade	Grade 3–5	Any grade	Grade 3–5	Any grade	Grade 3–5	Any grade	Grade 3–5
Treatment‐emergent AEs regardless of attribution, *n* (%)								
Overall	265 (88.3)	135 (45.0)	265 (91.4)	155 (53.4)	23 (79.3)	11 (37.9)	27 (100)	18 (66.7)
Serious	89 (29.7)	69 (23.0)	78 (26.9)	64 (22.1)	9 (31.0)	8 (27.6)	9 (33.3)	8 (29.6)
Led to discontinuation	26 (8.7)	20 (6.7)	15 (5.2)	11 (3.8)	1 (3.4)	1 (3.4)	3 (11.1)	2 (7.4)
Led to death	5 (1.7)	5 (1.7)	2 (0.7)	2 (0.7)	0	0	0	0
Treatment‐related AEs, *n* (%)								
Overall	170 (56.7)	44 (14.7)	236 (81.4)	117 (40.3)	14 (48.3)	2 (6.9)	26 (96.3)	15 (55.6)
Serious	26 (8.7)	19 (6.3)	34 (11.7)	28 (9.7)	2 (6.9)	1 (3.4)	3 (11.1)	2 (7.4)
Led to discontinuation	17 (5.7)	12 (4.0)	10 (3.4)	8 (2.8)	1 (3.4)	1 (3.4)	3 (11.1)	2 (7.4)
Led to death	0	0	2 (0.7)	2 (0.7)	0	0	0	0
Sponsor‐identified treatment‐emergent immune‐related AEs, *n* (%)								
Overall	47 (15.7)	16 (5.3)	2 (0.7)	2 (0.7)	7 (24.1)	1 (3.4)	0	0
Led to discontinuation	15 (5.0)	11 (3.7)	2 (0.7)	2 (0.7)	1 (3.4)	1 (3.4)	0	0
Led to death	0	0	0	0	0	0	0	0

Abbreviation: AE, adverse event.

^a^ Data cutoff date: January 4, 2021.

In the Japanese subgroup, the most common TEAEs of any grade in the cemiplimab treatment group were pyrexia (20.7%, *n* = 6) and insomnia (17.2%, *n* = 5); in the chemotherapy treatment group, the most common TEAEs of any grade were nausea (44.4%, *n* = 12) and anemia (40.7%, *n* = 11) (Table 4). The most common Grade ≥3 TEAEs in the cemiplimab treatment group were anemia (13.8%, *n* = 4) and urinary tract infection (6.9%, *n* = 2); the most common Grade ≥3 TEAEs in the chemotherapy treatment group were anemia (25.9%, *n* = 7), decreased neutrophil count (14.8%, *n* = 4), and decreased white blood cell count (14.8%, *n* = 4) (Table 4). Treatment‐related AEs (TRAEs) occurred in 48.3% (*n* = 14) of patients who received cemiplimab and 96.3% (*n* = 26) of patients who received chemotherapy; with events Grade ≥3 in 6.9% (*n* = 2) and 55.6% (*n* = 15) of patients, respectively (Table S2). Details of common TEAEs and TRAEs occurring in the overall population are provided in Tables S3 and S4, respectively.

**TABLE 4:** Treatment‐emergent AEs regardless of attribution in the Japanese subgroup^a^ ^,^ ^b^.

	Cemiplimab (*n* = 29)		Chemotherapy (*n* = 27)	
	Any grade	Grade 3–5	Any grade	Grade 3–5
Treatment‐emergent AEs, *n* (%)	23 (79.3)	11 (37.9)	27 (100)	18 (66.7)
Occurred in ≥10% of patients in either group, *n* (%)^c^				
Pyrexia	6 (20.7)	0	10 (37.0)	0
Insomnia	5 (17.2)	0	2 (7.4)	0
Anemia	4 (13.8)	4 (13.8)	11 (40.7)	7 (25.9)
Decreased appetite	4 (13.8)	1 (3.4)	7 (25.9)	2 (7.4)
Nausea	4 (13.8)	0	12 (44.4)	1 (3.7)
Back pain	3 (10.3)	0	1 (3.7)	0
Diarrhea	3 (10.3)	0	6 (22.2)	2 (7.4)
Hyperthyroidism	3 (10.3)	0	0	0
Hypothyroidism	3 (10.3)	0	0	0
Rash	3 (10.3)	0	1 (3.7)	0
Stomatitis	3 (10.3)	0	4 (14.8)	0
Hypoalbuminemia	2 (6.9)	0	3 (11.1)	2 (7.4)
Malaise	2 (6.9)	0	7 (25.9)	1 (3.7)
Urinary tract infection	2 (6.9)	2 (6.9)	3 (11.1)	3 (11.1)
Vomiting	2 (6.9)	0	6 (22.2)	0
Constipation	1 (3.4)	0	5 (18.5)	0
Decreased neutrophil count	1 (3.4)	1 (3.4)	9 (33.3)	4 (14.8)
Decreased platelet count	1 (3.4)	1 (3.4)	6 (22.2)	3 (11.1)
Decreased white blood cell count	1 (3.4)	0	5 (18.5)	4 (14.8)
Peripheral edema	1 (3.4)	0	3 (11.1)	0
Fatigue	0	0	3 (11.1)	0
Infusion‐related reaction	0	0	10 (37.0)	0

Abbreviation: AE, adverse event.

^a^ Data cutoff date: January 4, 2021.

^b^ Safety was assessed in all randomized patients who received ≥1 dose of the assigned treatment.

^c^ The events are listed in descending order of frequency in the cemiplimab treatment group. The events were coded according to the Preferred Terms of the Medical Dictionary for Regulatory Activities, version 23.1. The severity of AEs was graded according to the National Cancer Institute Common Terminology Criteria for Adverse Events, version 4.03.

In the Japanese subgroup, TEAEs of any grade leading to discontinuation occurred in 1 (3.4%) patient receiving cemiplimab (anemia, disseminated intravascular coagulation, hypothyroidism, abnormal hepatic function, increased amylase, decreased platelet count, and decreased appetite: *n* = 1 each) and 3 (11.1%) patients receiving chemotherapy (anemia, decreased appetite, malaise, anaphylactic reaction, infusion‐related reaction, and transient ischemic attack: *n* = 1 each) (Table S5). There were no TEAEs leading to death in the Japanese subgroup (Table 3).

### 3.4 Pharmacokinetics and immunogenicity

**FIGURE 3:**
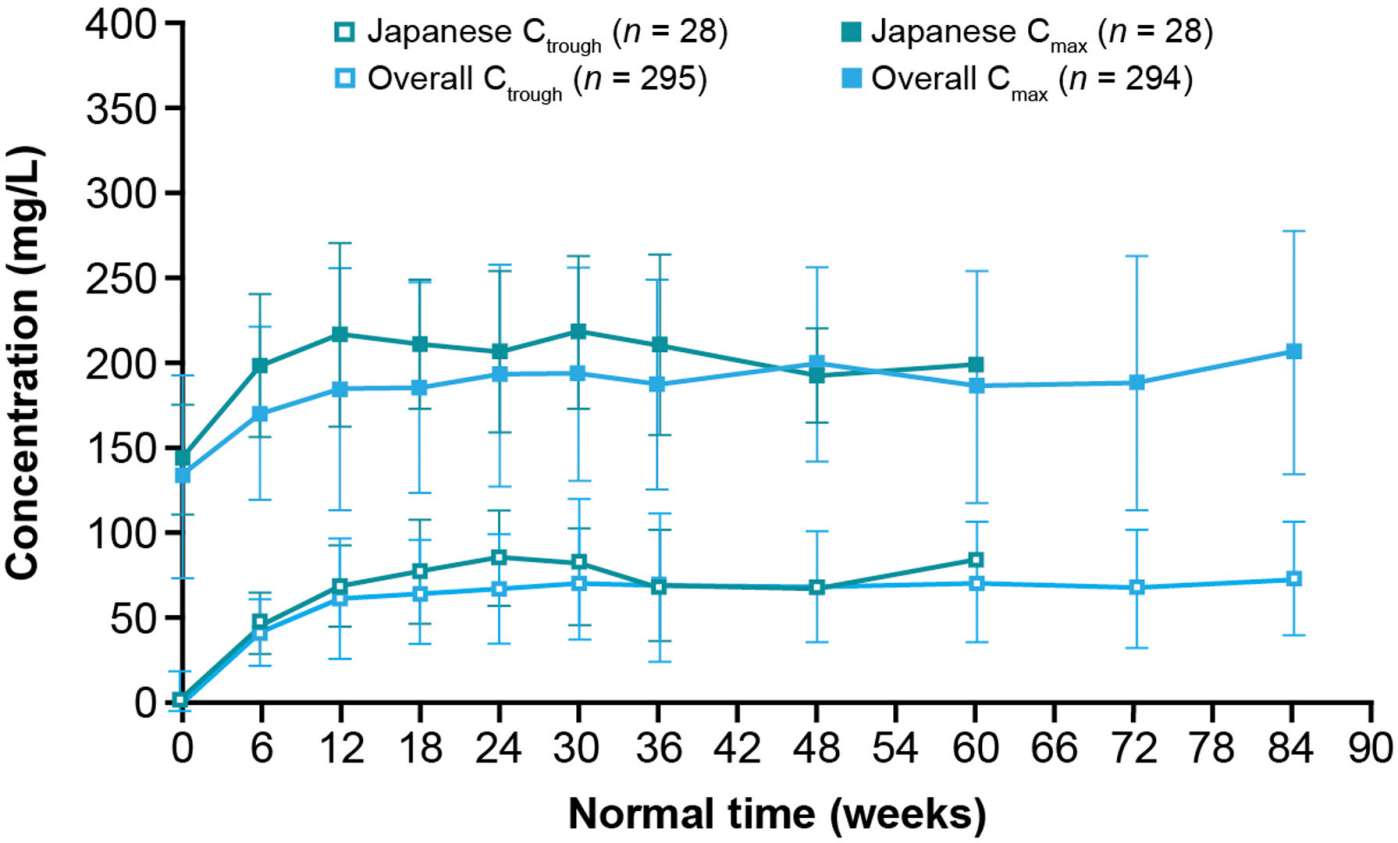
Observed mean (SD) cemiplimab *C*_trough_ and *C*_max_ over time in the Japanese subgroup and the overall study population treated with cemiplimab 350 mg every 3 weeks. Data cutoff date: January 4, 2021. *C*_max_, maximum concentration; *C*_trough_, trough concentration at the end of the dosing interval; SD, standard deviation.

Mean (standard deviation [SD]) trough concentration (*C*_trough_) of cemiplimab in serum at steady‐state was 78.4 (29.8) mg/L in the Japanese subgroup and 65.6 (30.0) mg/L in the overall population (Figure 3, Table S6). Mean (SD) maximum concentration (*C*_max_) of cemiplimab in serum at steady‐state in the Japanese subgroup was 211 (36.8) mg/L and 186 (60.8) mg/L in the overall population (Figure 3, Table S6). Although cemiplimab exposure metrics at steady‐state were approximately 13% (*C*_max_) to 20% (*C*_trough_) higher in the Japanese subgroup in than the overall population, this difference remains within the overall inter‐patient variability of cemiplimab exposure.

The ADA analysis set included 25 patients from the Japanese subgroup and 206 patients from the overall population. In the Japanese subgroup, 1 (4.0%) patient had treatment‐emergent ADAs and this was characterized as an indeterminate response and of low titer (<1000) (Table S7). In the overall population, treatment‐emergent ADAs were observed in 4 (1.9%) patients (Table S7). Of these 4 patients, 3 had indeterminate responses and 1 had a transient response; all were of low titer (<1000). Of the patients who developed treatment‐emergent antibodies to cemiplimab in the Japanese subgroup or the overall population, none developed neutralizing antibodies (Table S7). Moreover, no effect of ADAs on cemiplimab exposure was observed.

## 4 DISCUSSION

EMPOWER‐Cervical 1/GOG‐3016/ENGOT‐cx9 is the largest randomized study performed in patients with recurrent or metastatic cervical cancer who have experienced disease progression following platinum‐based chemotherapy. In this subgroup analysis of patients enrolled in Japan, cemiplimab monotherapy was associated with antitumor activity and had a positive benefit–risk profile. Treatment with cemiplimab reduced the risk of death by 14% and 31% in the Japanese subgroup and the overall population, respectively. This difference may be attributable to Japanese patients having a 4.6‐month‐shorter median duration of follow‐up compared with the overall population. Nevertheless, the observed HR of 0.86 in the Japanese subgroup suggests an emerging OS benefit with cemiplimab trending with the overall population. Cemiplimab treatment also resulted in a slightly longer PFS and a numerically higher ORR versus chemotherapy in the Japanese subgroup.

The overall safety profile of cemiplimab in the Japanese subgroup was consistent with the profile observed in the overall population,^30^ with no new safety signals identified. In both the Japanese subgroup and overall population, patients treated with cemiplimab had fewer AEs of any grade and fewer Grade ≥3 AEs than chemotherapy‐treated patients.

Pharmacokinetic analysis demonstrated that mean cemiplimab exposure metrics (*C*_trough_ and *C*_max_) at an intravenous dosage regimen of 350 mg Q3W were slightly higher in the Japanese subgroup compared with the overall trial population. The slightly higher mean exposures in the Japanese subgroup may be attributable to their lower median body weight compared with the overall trial population. However, as the differences in mean *C*_trough_ and *C*_max_ values between the Japanese subgroup and the overall trial population were within the overall inter‐patient variability of cemiplimab exposure, modification of cemiplimab dosage regimens is unnecessary for Japanese patients. Moreover, the effect of higher cemiplimab concentrations on antitumor activity and safety are likely to be minimal, given that relatively stable exposure–‐response relationships have been reported previously for the efficacy and safety of cemiplimab and other PD‐1 inhibitors.^53^, ^54^, ^55^

Both in Japan and worldwide, there is a high unmet need for effective second‐line therapies for the treatment of cervical cancer. Cemiplimab is the only single‐agent immunotherapy approved for second‐line treatment in the metastatic setting in Japan.^32^ As monotherapy, pembrolizumab is the only PD‐1 inhibitor approved in the United States for the second‐line treatment of recurrent or metastatic cervical cancer.^56^ However, in this setting, the approved use of pembrolizumab monotherapy is only permitted for patients whose tumors are PD‐L1‐positive with a combined positive score of ≥1, whereas cemiplimab monotherapy is approved in Japan, Brazil, Canada, and Europe irrespective of PD‐L1 expression status.^31^, ^32^, ^33^, ^34^, ^56^

Our post hoc analysis of the Japanese subgroup from EMPOWER‐Cervical 1/GOG‐3016/ENGOT‐cx9 has a few limitations. A comparatively smaller number of patients were enrolled in Japan, which limits the ability to make direct comparisons of the Japanese subgroup with the overall study population. Moreover, the small sample size of Japanese patients limits the ability of the current analysis to detect statistically significant differences in clinical efficacy (e.g., OS) between the cemiplimab and chemotherapy treatment arms of the Japanese subgroup. Despite these limitations, the antitumor activity and safety profile of cemiplimab observed in the Japanese subgroup of EMPOWER‐Cervical 1/GOG‐3016/ENGOT‐cx9 suggests cemiplimab is a potentially valuable treatment choice for Japanese patients with recurrent or metastatic cervical cancer.

## AUTHOR CONTRIBUTIONS

**Kosei Hasegawa:** Conceptualization (equal); formal analysis (equal); investigation (equal); writing – original draft (equal); writing – review and editing (equal). **Shunji Takahashi:** Data curation (equal); formal analysis (equal); writing – original draft (equal); writing – review and editing (equal). **Kimio Ushijima:** Data curation (equal); formal analysis (equal); writing – original draft (equal); writing – review and editing (equal). **Masao Okadome:** Data curation (equal); formal analysis (equal); writing – original draft (equal); writing – review and editing (equal). **Kan Yonemori:** Data curation (equal); formal analysis (equal); writing – original draft (equal); writing – review and editing (equal). **Harushige Yokota:** Data curation (equal); formal analysis (equal); writing – original draft (equal); writing – review and editing (equal). **Ignace Vergote:** Conceptualization (equal); data curation (equal); formal analysis (equal); writing – original draft (equal); writing – review and editing (equal). **Bradley J. Monk:** Conceptualization (equal); data curation (equal); formal analysis (equal); writing – original draft (equal); writing – review and editing (equal). **Krishnansu S. Tewari:** Data curation (equal); formal analysis (equal); writing – original draft (equal); writing – review and editing (equal). **Keiichi Fujiwara:** Data curation (equal); formal analysis (equal); writing – original draft (equal); writing – review and editing (equal). **Jingjin Li:** Conceptualization (equal); data curation (equal); formal analysis (equal); writing – original draft (equal); writing – review and editing (equal). **Shaheda Jamil:** Data curation (equal); formal analysis (equal); writing – original draft (equal); writing – review and editing (equal). **Anne Paccaly:** Conceptualization (equal); data curation (equal); formal analysis (equal); writing – original draft (equal); writing – review and editing (equal). **Kazuhiro Takehara:** Data curation (equal); formal analysis (equal); writing – original draft (equal); writing – review and editing (equal). **Tomoka Usami:** Data curation (equal); formal analysis (equal); writing – original draft (equal); writing – review and editing (equal). **Yoichi Aoki:** Data curation (equal); formal analysis (equal); writing – original draft (equal); writing – review and editing (equal). **Nao Suzuki:** Data curation (equal); formal analysis (equal); writing – original draft (equal); writing – review and editing (equal). **Yoichi Kobayashi:** Data curation (equal); formal analysis (equal); writing – original draft (equal); writing – review and editing (equal). **Yoshio Yoshida:** Data curation (equal); formal analysis (equal); writing – original draft (equal); writing – review and editing (equal). **Hidemichi Watari:** Data curation (equal); formal analysis (equal); writing – original draft (equal); writing – review and editing (equal). **Frank Seebach:** Conceptualization (equal); data curation (equal); formal analysis (equal); writing – original draft (equal); writing – review and editing (equal). **Israel Lowy:** Conceptualization (equal); data curation (equal); formal analysis (equal); writing – original draft (equal); writing – review and editing (equal). **Melissa Mathias:** Conceptualization (equal); data curation (equal); formal analysis (equal); writing – original draft (equal); writing – review and editing (equal). **Matthew G. Fury:** Conceptualization (equal); data curation (equal); formal analysis (equal); writing – original draft (equal); writing – review and editing (equal). **Ana Oaknin:** Conceptualization (equal); data curation (equal); formal analysis (equal); writing – original draft (equal); writing – review and editing (equal).

## FUNDING INFORMATION

This study was funded by Regeneron Pharmaceuticals, Inc., and Sanofi.

## CONFLICT OF INTEREST STATEMENT

Kosei Hasegawa reports research grants from Merck Sharp & Dohme, Ono, Takeda, Daiichi‐Sankyo, and Eisai; honoraria from Takeda, Chugai Pharma, Kyowa‐Kirin, Genmab, AstraZeneca, and Merck Sharp & Dohme; and consulting/advisory board fees from Merck Sharp & Dohme, Eisai, and Takeda. Shunji Takahashi reports honoraria from Daiichi Sankyo, Eisai, Bayer, Taiho Pharmaceutical, Merck Sharp & Dohme, Novartis, Chugai Pharma, AstraZeneca, Bristol‐Myers Squibb Japan, Ono Pharmaceutical, Nihonkayaku, Pfizer, and Lilly Japan; an advisory role at Bayer; research funding from Daiichi Sankyo, Sanofi, Eisai, Bayer, Taiho Pharmaceutical, Merck Sharp & Dohme, Novartis, Chugai Pharma, AstraZeneca, Bristol‐Myers Squibb, Lilly, Ono Pharmaceutical, PharmaMar, and Pfizer/EMD Serono; and travel, accommodation, and expenses from Daiichi Sankyo and Novartis. Kimio Ushijima reports honoraria from Chugai Pharma; and research funding from Kaken Pharmaceutical, Takeda Pharmaceutical Company, and Tsumura & Co. Masao Okadome reports no conflict of interest. Kan Yonemori reports lecture fees and/or advisory fees from AstraZeneca, Chugai Pharma, Daiichi Sankyo, Esai, Ono Pharmaceutical, Novartis, Pfizer, and Takeda Pharmaceutical Company. Harushige Yokota reports honoraria from Chugai Pharma, Kaken Pharmaceutical, and Merck Sharp & Dohme; and research funding from Pfizer and Zeria Pharmaceutical. Ignace Vergote reports consulting fees from AstraZeneca, Elevar Therapeutics, Genmab, GlaxoSmithKline, Immunogen, Merck Sharp & Dohme, and Oncoinvent; and contracted research from Genmab and Hoffmann‐La Roche. Bradley J Monk reports consulting honoraria from Aravive, Asymmetric Therapeutics, Boston Biomedical, ChemoCare, ChemoID, Circulogene, Conjupro Biotherapeutics, Eisai, Geistlich, Genmab/Seattle Genetics, Gynecologic Oncology Group Foundation, ImmunoGen, Immunomedics, Incyte, Laekna Health Care, Mateon/Oxigene, Merck, Mersana, Myriad, Nucana, Oncomed, Oncoquest, Oncosec, Perthera, Pfizer, Precision Oncology, Puma, Regeneron, Samumed, Takeda, VBL, and Vigeo; and consulting/speaker honoraria from AstraZeneca, Clovis, Janssen/Johnson & Johnson, Roche/Genentech, and Tesaro/GSK. Krishnansu S Tewari reports honoraria from Tesaro and Clovis Oncology; consulting fees from Genentech, Tesaro, Clovis, and AstraZeneca; speaker fees from Genentech, AstraZeneca, Merck, Tesaro, and Clovis; research grants from AbbVie, Genentech, Morphotek, Merck, and Regeneron Pharmaceuticals, Inc; and travel/accommodation expenses from Genentech. Keiichi Fujiwara reports receiving consulting fees and/or grant support from Pfizer, Daiichi Sankyo, Eisai, Immunogen, Kyowa Hakko Kirin, Merck Sharp & Dohme, Mochida Pharmaceutical, NanoCarrier, Novartis, Oncotherapy, Regeneron Pharmaceuticals, Inc., Taiho, Zeria, Chugai Pharma, Genmab, and Takeda Pharmaceutical Company. Jingjin Li, Shaheda Jamil, Anne Paccaly, Frank Seebach, Israel Lowy, Melissa Mathias, and Matthew G Fury are employees and shareholders of Regeneron Pharmaceuticals, Inc. Kazuhiro Takehara reports lecture fees and consulting fees from Takeda. Tomoka Usami reports no conflict of interest. Yoichi Aoki reports no conflict of interest. Nao Suzuki reports no conflict of interest. Yoichi Kobayashi reports no conflict of interest. Yoshio Yoshida reports no conflict of interest. Hidemichi Watari reports honoraria from Merck Sharp & Dohme, Tsumura & Co, AstraZeneca, Aska Pharmaceutical Co, Ltd, Terumo, Bayer Yakuhin, Kaken Pharmaceutical, Mochida Pharmaceutical Co, Ltd, Daiichi Sankyo/UCB Japan, Chugai Pharmaceutical, and Takeda; consulting/advisory board fees from Decision Resources Group Japan, Kaken Pharmaceutical, and Chugai Pharmaceutical; and research grants from Hokkaido Welfare Federation of Agricultural Cooperatives, Kaken Pharmaceutical, Mochida Pharmaceutical Co, Ltd, Taiho Pharmaceutical, Chugai Pharmaceutical, Hokkaido Cancer Society, and Takeda. Ana Oaknin reports advisory board fees from Roche, AstraZeneca, PharmaMar, Clovis Oncology, Tesaro, Inmunogen, Genmab, Mersana Therapeutic, GlaxoSmithKline, and Deciphera Pharmaceuticals; and support for travel or accommodation from Roche, AstraZeneca, and PharmaMar.

## ETHICS STATEMENT

*Approval of the research protocol*: The study protocol and all amendments were approved by the institutional review board of Saitama Medical University International Medical Center (approval number: 208), along with all necessary institutional and jurisdictional ethics committees. The study was conducted in accordance with the principles of the Declaration of Helsinki and the International Conference on Harmonization Good Clinical Practice guidelines. *Informed consent*: All patients provided written informed consent before enrollment.*Registry and registration no. of the study/trial*: The trial was registered at CllinicalTrials.gov: NCT03257267. *Animal studies*: N/A.

## DISCLOSURES

None of the authors of this manuscript is a current Editor or Editorial Board Member of Cancer Science.

## Supporting information


Table S1.

## Data Availability

Qualified researchers may request access to study documents (including the clinical study report, study protocol with any amendments, blank case report form, statistical analysis plan) that support the methods and findings reported in this manuscript. Individual anonymized participant data will be considered for sharing once the product and indication has been approved by major health authorities (e.g., FDA, EMA, PMDA, etc), if there is legal authority to share the data and there is not a reasonable likelihood of participant re‐identification. Submit requests to https://vivli.org/.
