# Supplementary material for: Cemiplimab monotherapy in Japanese patients with recurrent or metastatic cervical cancer
Source: Cancer Med. 2024 Sep 26;13(18):e70236. doi: 10.1002/cam4.70236 (PMC11426160; doi:10.1002/cam4.70236)
Supplement: Supplementary file 1 — Table S1. [file CAM4-13-e70236-s001.docx]

# Supplementary Table 1. Investigator’s choice of chemotherapy among randomized patients in the Japanese subgroup and the overall study population^a^

|  | **Overall study population** | **Japan subgroup** |
| --- | --- | --- |
| **Investigator’s choice of chemotherapy, *n* (%)^b^** | **Chemotherapy (*n* = 290)** | **Chemotherapy (*n* = 27)** |
| Gemcitabine | 118 (40.7) | 19 (70.4) |
| Irinotecan | 19 (6.5) | 8 (29.6) |
| Pemetrexed | 109 (37.6) | 0 |
| Topotecan | 16 (5.5) | 0 |
| Vinorelbine | 28 (9.7) | 0 |

^a^Data cutoff date: January 4, 2021.

^b^The selection of single-agent chemotherapy was determined prior to randomization by the investigator from protocol-specified options.

# Supplementary Table 2. Treatment-related AEs in the Japanese subgroup^a,b^

|  | **Cemiplimab**  **(*n* = 29)** | | **Chemotherapy**  **(*n* = 27)** | |
| --- | --- | --- | --- | --- |
| **Event** | **Any grade** | **Grade 3-5** | **Any grade** | **Grade 3-5** |
| Treatment-related AEs, *n* (%) | 14 (48.3) | 2 (6.9) | 26 (96.3) | 15 (55.6) |
| Occurred in ≥10% of patients in either group, *n* (%)^c^ |  |  |  |  |
| Decreased appetite | 3 (10.3) | 0 | 5 (18.5) | 1 (3.7) |
| Hyperthyroidism | 3 (10.3) | 0 | 0 | 0 |
| Hypothyroidism | 3 (10.3) | 0 | 0 | 0 |
| Rash | 3 (10.3) | 0 | 1 (3.7) | 0 |
| Stomatitis | 3 (10.3) | 0 | 4 (14.8) | 0 |
| Anemia | 2 (6.9) | 2 (6.9) | 9 (33.3) | 6 (22.2) |
| Malaise | 2 (6.9) | 0 | 6 (22.2) | 0 |
| Pyrexia | 2 (6.9) | 0 | 5 (18.5) | 0 |
| Decreased platelet count | 1 (3.4) | 1 (3.4) | 6 (22.2) | 3 (11.1) |
| Diarrhea | 1 (3.4) | 0 | 5 (18.5) | 2 (7.4) |
| Nausea | 1 (3.4) | 0 | 11 (40.7) | 1 (3.7) |
| Constipation | 0 | 0 | 3 (11.1) | 0 |
| Decreased neutrophil count | 0 | 0 | 9 (33.3) | 4 (14.8) |
| Decreased white blood cell count | 0 | 0 | 5 (18.5) | 4 (14.8) |
| Fatigue | 0 | 0 | 3 (11.1) | 0 |
| Infusion related reaction | 0 | 0 | 10 (37.0) | 0 |
| Vomiting | 0 | 0 | 6 (22.2) | 0 |

^a^Data cutoff date: January 4, 2021.

^b^Safety was assessed in all randomized patients who received ≥1 dose of the assigned treatment.

^c^The events are listed in descending order of frequency in the cemiplimab treatment group. The events were coded according to the Preferred Terms of the Medical Dictionary for Regulatory Activities, version 23.1. The severity of AEs was graded according to the National Cancer Institute Common Terminology Criteria for Adverse Events, version 4.03.

AE, adverse event.

# Supplementary Table 3. Treatment-emergent AEs regardless of attribution in the overall study population^a,b^

|  | **Cemiplimab**  **(*n* = 300)** | | **Chemotherapy**  **(*n* = 290)** | |
| --- | --- | --- | --- | --- |
| **Event** | **Any grade** | **Grade 3-5** | **Any grade** | **Grade 3-5** |
| Treatment-emergent AEs, *n* (%) | 265 (88.3) | 135 (45.0) | 265 (91.4) | 155 (53.4) |
| Occurred in ≥10% of patients in either group, *n* (%)^c^ |  |  |  |  |
| Anemia | 75 (25.0) | 36 (12.0) | 129 (44.5) | 78 (26.9) |
| Nausea | 55 (18.3) | 1 (0.3) | 97 (33.4) | 6 (2.1) |
| Fatigue | 50 (16.7) | 4 (1.3) | 45 (15.5) | 4 (1.4) |
| Vomiting | 48 (16.0) | 2 (0.7) | 68 (23.4) | 7 (2.4) |
| Decreased appetite | 45 (15.0) | 1 (0.3) | 46 (15.9) | 2 (0.7) |
| Constipation | 45 (15.0) | 0 | 59 (20.3) | 1 (0.3) |
| Pyrexia | 35 (11.7) | 1 (0.3) | 61 (21.0) | 0 |
| Urinary tract infection | 35 (11.7) | 15 (5.0) | 25 (8.6) | 8 (2.8) |
| Asthenia | 33 (11.0) | 7 (2.3) | 44 (15.2) | 3 (1.0) |
| Back pain | 33 (11.0) | 4 (1.3) | 25 (8.6) | 2 (0.7) |
| Diarrhea | 32 (10.7) | 3 (1.0) | 39 (13.4) | 4 (1.4) |
| Arthralgia | 31 (10.3) | 1 (0.3) | 8 (2.8) | 0 |
| Abdominal pain | 29 (9.7) | 3 (1.0) | 33 (11.4) | 3 (1.0) |
| Neutropenia | 6 (2.0) | 3 (1.0) | 44 (15.2) | 26 (9.0) |

^a^Data cutoff date: January 4, 2021.

^b^Safety was assessed in all randomized patients who received ≥1 dose of the assigned treatment.

^c^The events are listed in descending order of frequency in the cemiplimab treatment group. The events were coded according to the Preferred Terms of the Medical Dictionary for Regulatory Activities, version 23.1. The severity of AEs was graded according to the National Cancer Institute Common Terminology Criteria for Adverse Events, version 4.03.

AE, adverse event.

# Supplementary Table 4. Treatment-related AEs in the overall study population^a,b^

|  | **Cemiplimab**  **(*n* = 300)** | | **Chemotherapy**  **(*n* = 290)** | |
| --- | --- | --- | --- | --- |
| **Event** | **Any grade** | **Grade 3-5** | **Any grade** | **Grade 3-5** |
| Treatment-related AEs, *n* (%) | 170 (56.7) | 44 (14.7) | 236 (81.4) | 117 (40.3) |
| Occurred in ≥10% of patients in either group, *n* (%)^c^ |  |  |  |  |
| Fatigue | 32 (10.7) | 2 (0.7) | 39 (13.4) | 3 (1.0) |
| Nausea | 28 (9.3) | 0 | 87 (30.0) | 3 (1.0) |
| Anemia | 22 (7.3) | 11 (3.7) | 106 (36.6) | 62 (21.4) |
| Asthenia | 22 (7.3) | 2 (0.7) | 33 (11.4) | 2 (0.7) |
| Vomiting | 17 (5.7) | 0 | 53 (18.3) | 4 (1.4) |
| Pyrexia | 8 (2.7) | 1 (0.3) | 29 (10.0) | 0 |
| Neutropenia | 6 (2.0) | 3 (1.0) | 43 (14.8) | 25 (8.6) |

^a^Data cutoff date: January 4, 2021.

^b^Safety was assessed in all randomized patients who received ≥1 dose of the assigned treatment.^\^

^c^The events are listed in descending order of frequency in the cemiplimab treatment group. The events were coded according to the Preferred Terms of the Medical Dictionary for Regulatory Activities, version 23.1. The severity of AEs was graded according to the National Cancer Institute Common Terminology Criteria for Adverse Events, version 4.03.

AE, adverse event.

# Supplementary Table 5. Treatment-emergent AEs leading to treatment discontinuation in the Japanese subgroup^a,b^

|  | **Cemiplimab**  **(*n* = 29)** | | **Chemotherapy**  **(*n* = 27)** | |
| --- | --- | --- | --- | --- |
| **Event** | **Any grade** | **Grade 3-5** | **Any grade** | **Grade 3-5** |
| Treatment-emergent AEs leading to discontinuation, *n* (%) | 1 (3.4) | 1 (3.4) | 3 (11.1) | 2 (7.4%) |
| Abnormal hepatic function | 1 (3.4) | 1 (3.4) | 0 | 0 |
| Anemia | 1 (3.4) | 1 (3.4) | 1 (3.7) | 1 (3.7) |
| Decreased appetite | 1 (3.4) | 0 | 1 (3.7) | 1 (3.7) |
| Decreased platelet count | 1 (3.4) | 1 (3.4) | 0 | 0 |
| Disseminated intravascular coagulation | 1 (3.4) | 0 | 0 | 0 |
| Hypothyroidism | 1 (3.4) | 0 | 0 | 0 |
| Increased amylase | 1 (3.4) | 1 (3.4) | 0 | 0 |
| Anaphylactic reaction | 0 | 0 | 1 (3.7) | 1 (3.7) |
| Infusion-related reaction | 0 | 0 | 1 (3.7) | 0 |
| Malaise | 0 | 0 | 1 (3.7) | 1 (3.7) |
| Transient ischemic attack | 0 | 0 | 1 (3.7) | 1 (3.7) |

^a^Data cutoff date: January 4, 2021.

^b^Safety was assessed in all randomized patients who received ≥1 dose of the assigned treatment.^\^

^c^The events are listed in descending order of frequency in the cemiplimab treatment group. The events were coded according to the Preferred Terms of the Medical Dictionary for Regulatory Activities, version 23.1. The severity of AEs was graded according to the National Cancer Institute Common Terminology Criteria for Adverse Events, version 4.03.

AE, adverse event.

# Supplementary Table 6. Observed mean (SD) cemiplimab C_trough_ and C_max_ after the first dose and at steady-state in the Japanese subgroup and the overall study population treated with cemiplimab 350 mg every 3 weeks^a^

|  | **After the first dose** | | | | | | **At steady-state (assessed on week 18)** | | | | | |
| --- | --- | --- | --- | --- | --- | --- | --- | --- | --- | --- | --- | --- |
|  | **C_trough_, mg/L** | | | **C_max_, mg/L** | | | **C_trough_, mg/L** | | | **C_max_, mg/L** | | |
| **=** | ***n*** | **Mean  (SD)** | **Median (IQR)** | ***n*** | **Mean  (SD)** | **Median (IQR)** | ***n*** | **Mean  (SD)** | **Median (IQR)** | ***n*** | **Mean  (SD)** | **Median (IQR)** |
| Overall study population (*n* = 295) | NA | NA | NA | 284 | 134  (58.7) | 130 (108-156) | 113 | 65.6  (30.0) | 60.7 (46.4-82.9) | 112 | 186 (60.8) | 189 (149-217) |
| Japanese subgroup (*n* = 28) | NA | NA | NA | 28 | 144  (32.1) | 143  (124-159) | 13 | 78.4 (29.8) | 77.1  (62.4-95.3) | 12 | 211 (36.8) | 213 (194-224) |

^a^Data cutoff date: January 4, 2021.

C_max_, maximum concentration; C_trough_, trough concentration at the end of the dosing interval; IQR, interquartile range; NA, not applicable; SD, standard deviation.

# Supplementary Table 7. Summary of ADA status and ADA category in the Japanese subgroup and the overall study population^a^

| **ADA status and category** | **Overall study population** | **Japanese  subgroup** |
| --- | --- | --- |
| Total patients with ADAs, *n* (%) | 206 (100) | 25 (100) |
| Negative, *n* (%) | 196 (95.1) | 24 (96.0) |
| Pre-existing, *n* (%) | 6 (2.9) | 0 |
| Treatment-boosted response, *n* (%) | 0 | 0 |
| Treatment-emergent response, *n* (%) | 4 (1.9) | 1 (4.0) |
| Treatment-emergent and treatment-boosted category, *n* (%) |  |  |
| Persistent | 0 | 0 |
| Transient | 1 (0.5) | 0 |
| Indeterminate | 3 (1.5) | 1 (4.0) |
| Treatment-emergent and treatment-boosted maximum titer category, *n* (%) |  |  |
| Low (<1000) | 4 (1.9) | 1 (4.0) |
| Moderate (1000 to 10,000) | 0 | 0 |
| High (>10,000) | 0 | 0 |
| Treatment-emergent and treatment-boosted neutralizing antibody status, *n* (%) |  |  |
| Neutralizing antibody negative | 4 (1.9) | 1 (4.0) |
| Neutralizing antibody positive | 0 | 0 |

^a^Data cutoff date: January 4, 2021.

ADA, antidrug antibody.
